# Supplementary material for: Neuregulin (NRG-1β) Is Pro-Myogenic and Anti-Cachectic in Respiratory Muscles of Post-Myocardial Infarcted Swine
Source: Biology (Basel). 2022 Apr 29;11(5):682. doi: 10.3390/biology11050682 (PMC9137990; doi:10.3390/biology11050682)
Supplement: Supplementary file 1 [file biology-11-00682-s001.zip › Supplementary Figure S1.pdf]

Appendix B: Supplementary Figures

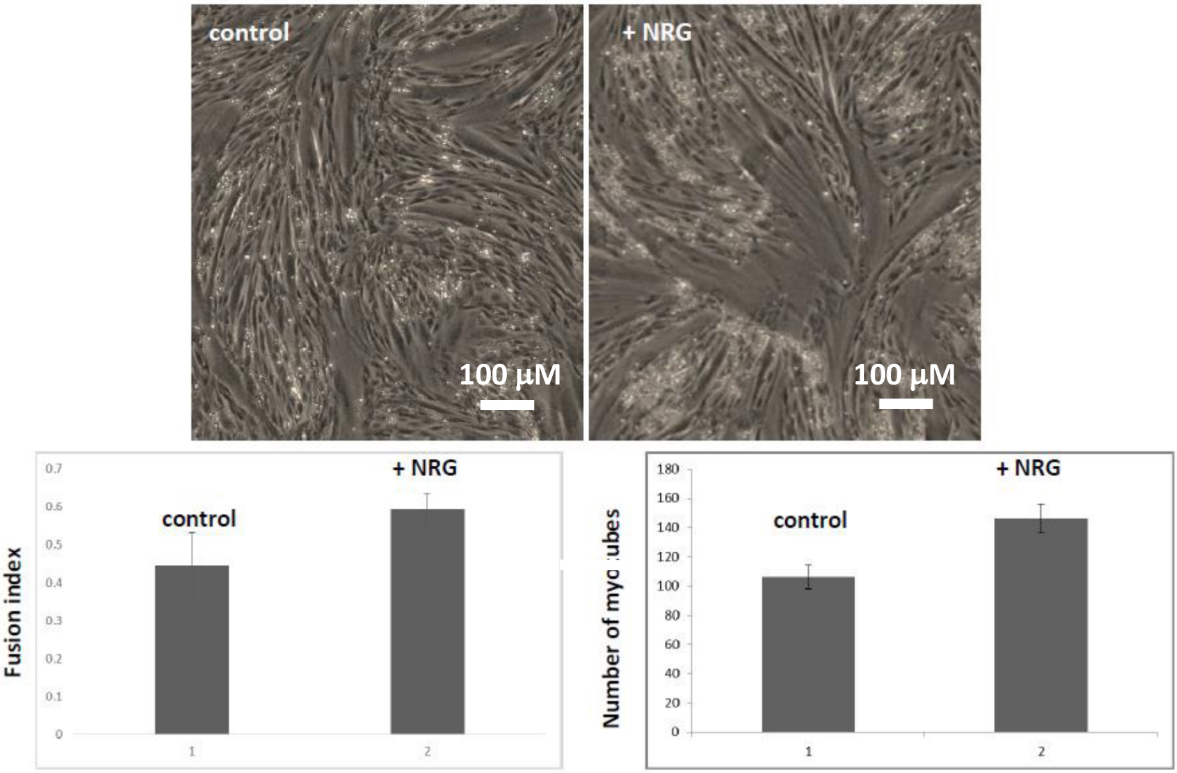

Figure S1: L6 myoblasts were differentiated in DMEM with 0.2% BSA in the absence or treatment (control, tope left) or with neuregulin (+ NRG, top right). The cells were stained, as described in the Materials and Methods section, and the fusion index (bottom left) and number of myotubes (bottom right) were determined, as shown graphically.
