# Supplementary material for: Neuregulin (NRG-1β) Is Pro-Myogenic and Anti-Cachectic in Respiratory Muscles of Post-Myocardial Infarcted Swine
Source: Biology (Basel). 2022 Apr 29;11(5):682. doi: 10.3390/biology11050682 (PMC9137990; doi:10.3390/biology11050682)
Supplement: Supplementary file 1 [file biology-11-00682-s001.zip › Supplementary Figure S2.pdf]

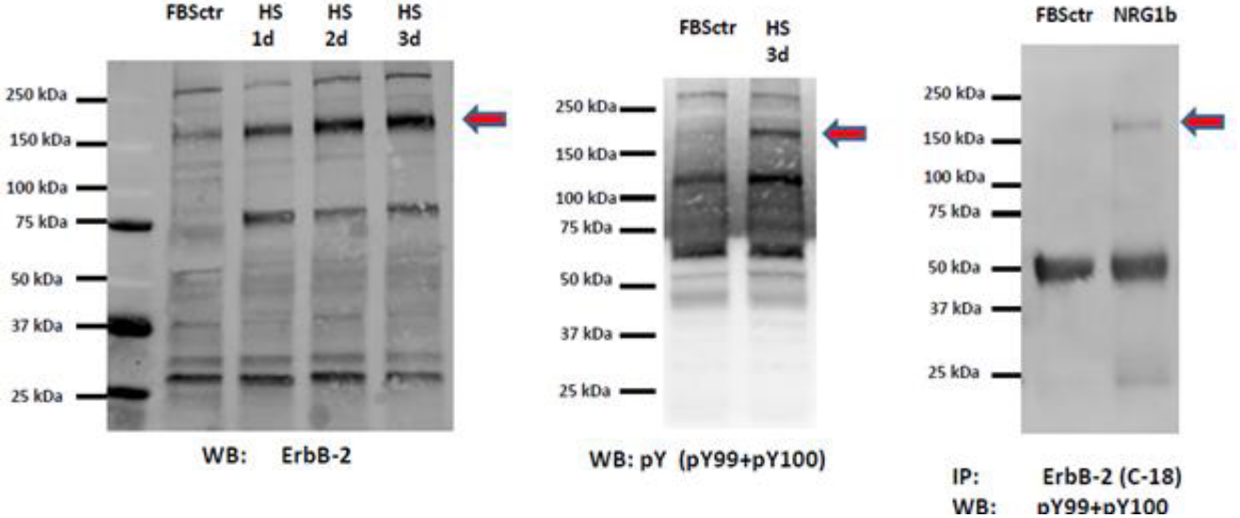

Figure S2: C2C12 cells were differentiated in DMEM with 2% horse serum (HS) and protein lysates collected at first three days (day 1= **1d**, day 2=**2d**, day 3 = **3d**) and subjected to gel electrophoresis and Western blotting using an antibody against pan ErbB2 (Left side) or against erbB2 phosphorylated at tyrosine 99 (pY99) and pY100 (middle). Cells grown in growth media (DMEM with fetal bovine serum) served as a control (**FBSctr)** As shown (Left side), ErbB2 was increased beginning at day 1 and increased further to day 3. ErbB2 in day 3 lysates was also phosphorylated (middle). Day 3 Lysates were also subjected to pull down assay (IP) using an antibody against neuregulin 1-β (NRG1b, right side) and Western blotting (WB) performed using ErbB2 antibody (right side). Arrows indicate band at expected size (185 kDa),

.
