# Supplementary material for: Neuregulin (NRG-1β) Is Pro-Myogenic and Anti-Cachectic in Respiratory Muscles of Post-Myocardial Infarcted Swine
Source: Biology (Basel). 2022 Apr 29;11(5):682. doi: 10.3390/biology11050682 (PMC9137990; doi:10.3390/biology11050682)
Supplement: Supplementary file 1 [file biology-11-00682-s001.zip › Supplementary Figure S3.pdf]

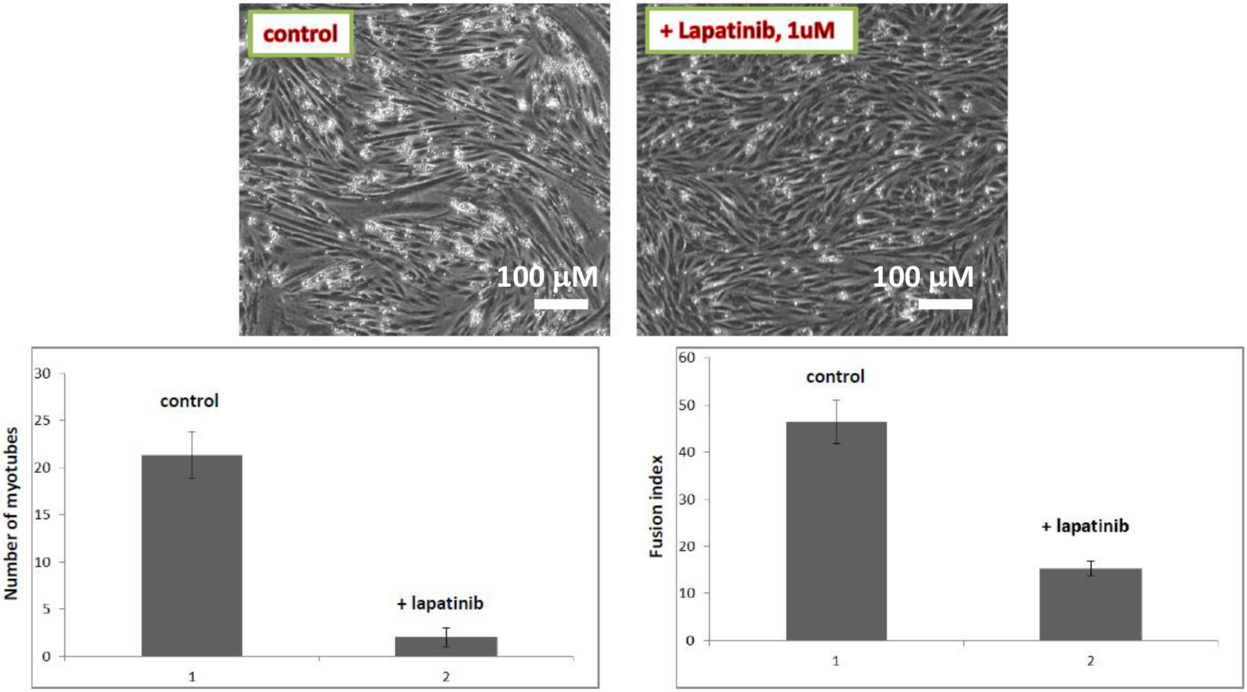

Figure S3: L6 cells were differentiated in DMEM with 0.2% BSA in the presence of 1 uM of the ErbB2 inhibitor, lapatinib (top right), which inhibited myotube formation, as measured by number of myotubes (bottom left) and fusion index (bottom right).
