# Supplementary material for: Neuregulin (NRG-1β) Is Pro-Myogenic and Anti-Cachectic in Respiratory Muscles of Post-Myocardial Infarcted Swine
Source: Biology (Basel). 2022 Apr 29;11(5):682. doi: 10.3390/biology11050682 (PMC9137990; doi:10.3390/biology11050682)
Supplement: Supplementary file 1 [file biology-11-00682-s001.zip › Supplementary Figure S4.pdf]

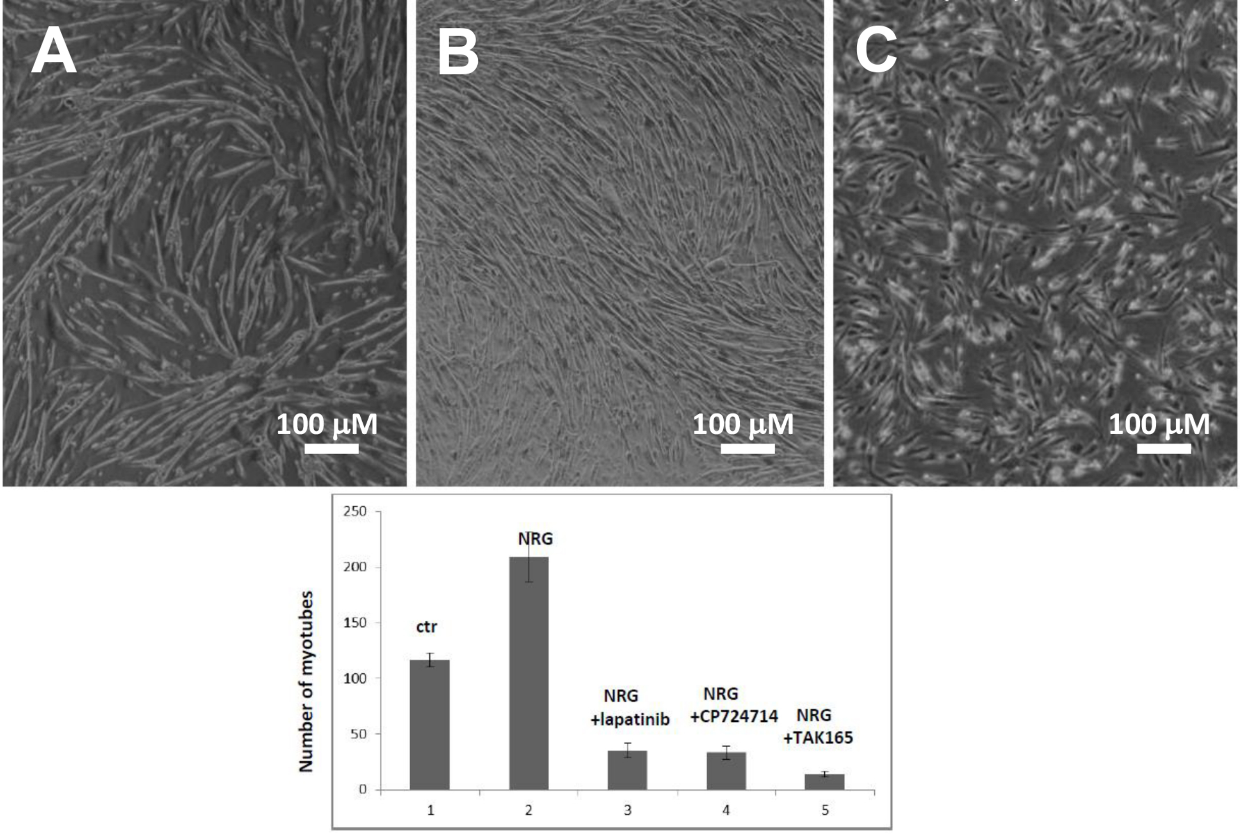

Figure S4: C2C12 myoblasts were differentiated in the presence or absence of NRG and 1uM lapatinib, 2 uM of CP724714 or 0.2 uM TAK165. Representative images of control cells (A), cells treated with NRG only (B) and NRG + lapatinib (C) are shown. Number of myotubes after treatment with NRG in the presence or absence of the various inhibitors are shown graphically. Ctr = untreated control
