# Supplementary material for: Neuregulin (NRG-1β) Is Pro-Myogenic and Anti-Cachectic in Respiratory Muscles of Post-Myocardial Infarcted Swine
Source: Biology (Basel). 2022 Apr 29;11(5):682. doi: 10.3390/biology11050682 (PMC9137990; doi:10.3390/biology11050682)
Supplement: Supplementary file 1 [file biology-11-00682-s001.zip › Supplementary Figure S5.pdf]

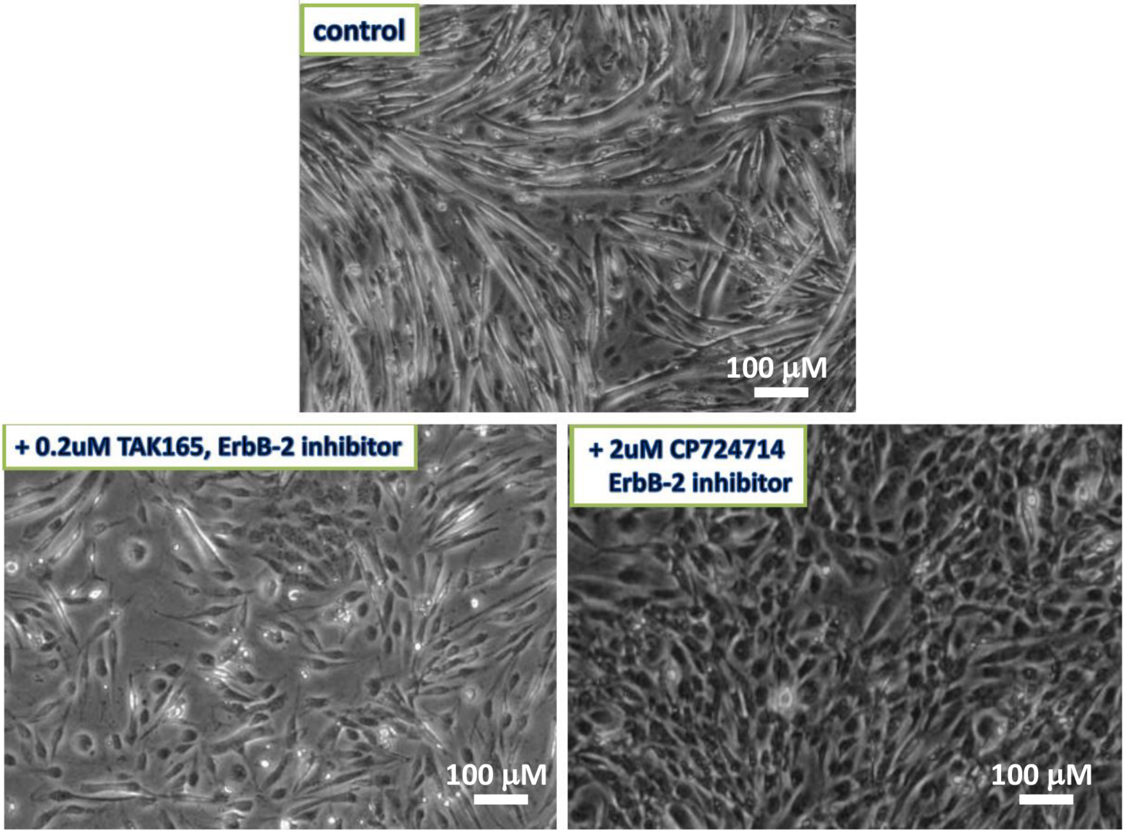

Figure S5: C2C12 myoblasts were differentiated in DMEM with 2% horse serum in the presence or absence of 2 uM of CP724714 or 0.2 uM TAK165. Representative images of control cells (top left) and cells treated with the inhibitors are shown.
