# Supplementary material for: Neuregulin (NRG-1β) Is Pro-Myogenic and Anti-Cachectic in Respiratory Muscles of Post-Myocardial Infarcted Swine
Source: Biology (Basel). 2022 Apr 29;11(5):682. doi: 10.3390/biology11050682 (PMC9137990; doi:10.3390/biology11050682)
Supplement: Supplementary file 1 [file biology-11-00682-s001.zip › Supplementary Figure S6.pdf]

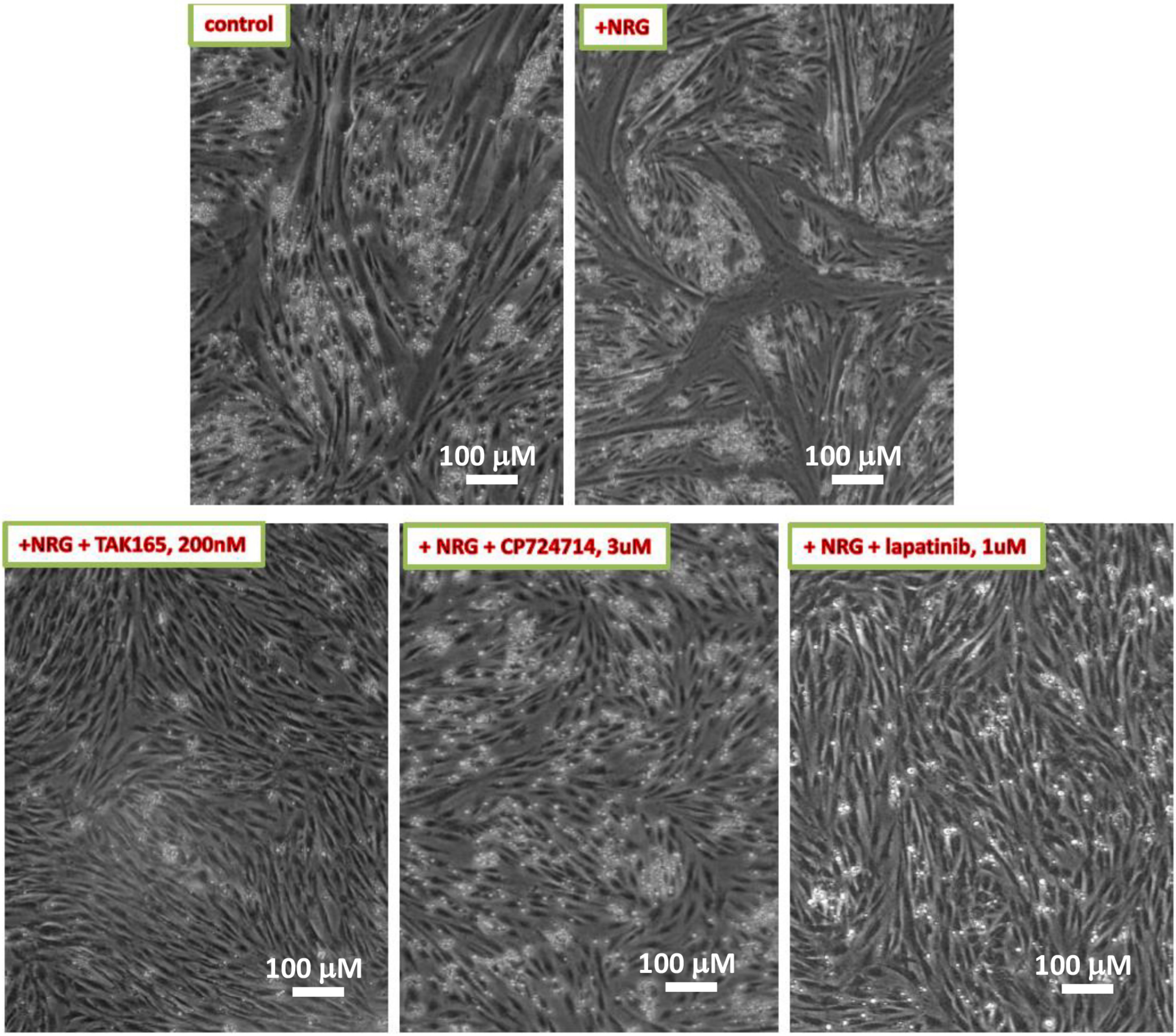

Figure S6: L6 cells were differentiated in DMEM with 0.2% BSA in the absence (control, top left) or presence of NRG with 0.2 uM TAK165, 3 uM of CP724714 or 1uM lapatinib.
