# Supplementary material for: Neuregulin (NRG-1β) Is Pro-Myogenic and Anti-Cachectic in Respiratory Muscles of Post-Myocardial Infarcted Swine
Source: Biology (Basel). 2022 Apr 29;11(5):682. doi: 10.3390/biology11050682 (PMC9137990; doi:10.3390/biology11050682)
Supplement: Supplementary file 1 [file biology-11-00682-s001.zip › Supplementary Figure S7.pdf]

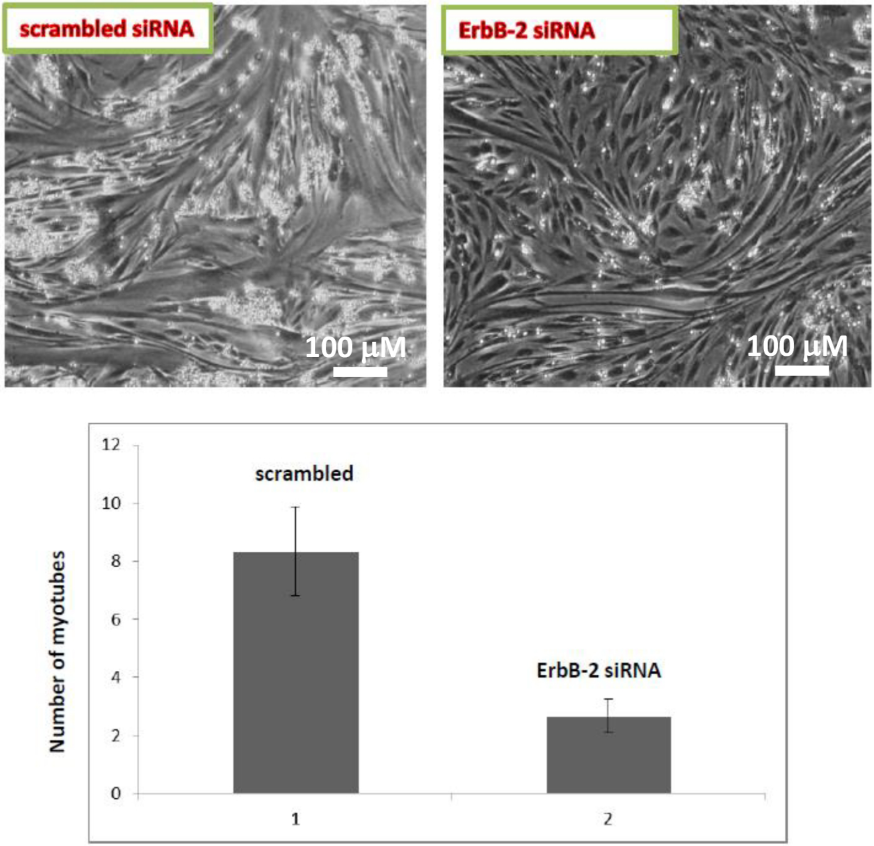

Figure S7: L6 cells were transfected with ErbB-2-specific siRNA or scrambled siRNA as a control and then differentiated in DMEM without serum. In cells transfected with ErbB-2 siRNA, the levels of ErbB-2 were significantly downregulated (Top right), compared to control (top left) as depicted graphically (bottom).
