# Supplementary figures and images for: Neuregulin (NRG-1β) Is Pro-Myogenic and Anti-Cachectic in Respiratory Muscles of Post-Myocardial Infarcted Swine
Source: Biology (Basel). 2022 Apr 29;11(5):682. doi: 10.3390/biology11050682 (PMC9137990; doi:10.3390/biology11050682)

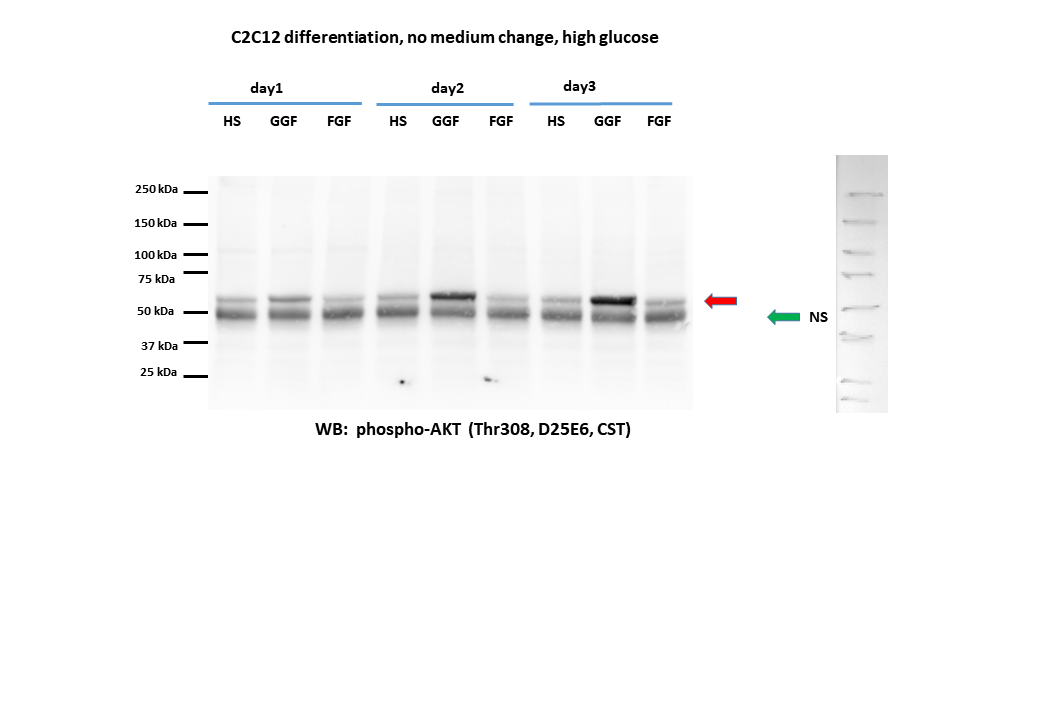

Supplement: Supplementary file 1 [file biology-11-00682-s001.zip › Figure S10.PNG]

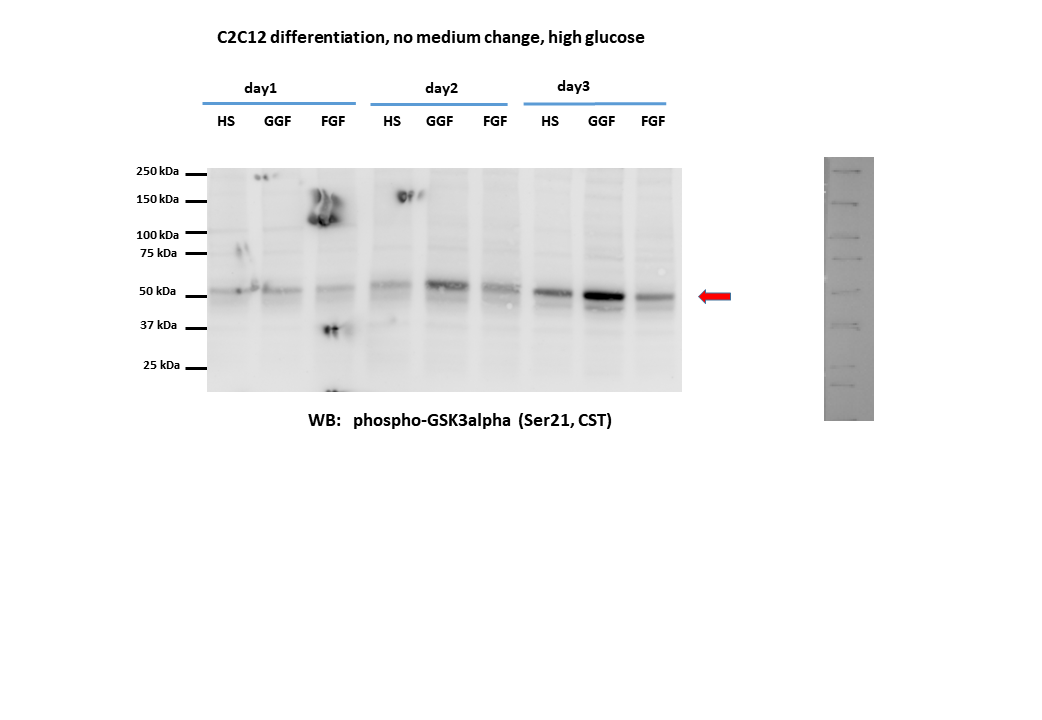

Supplement: Supplementary file 1 [file biology-11-00682-s001.zip › Figure S11.PNG]

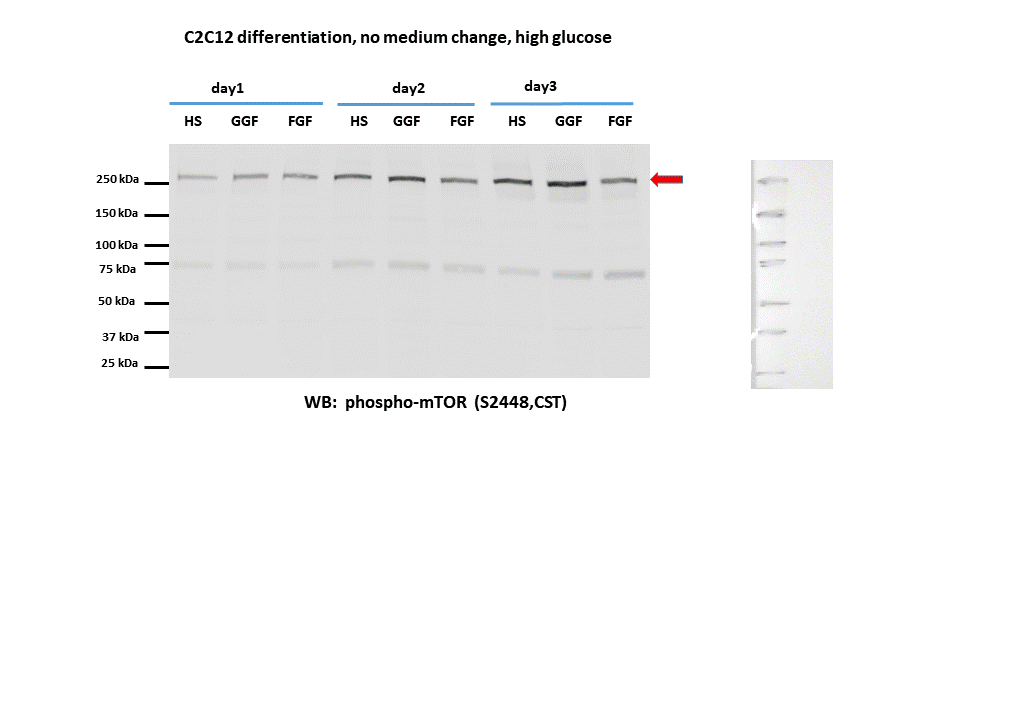

Supplement: Supplementary file 1 [file biology-11-00682-s001.zip › Figure S12.PNG]

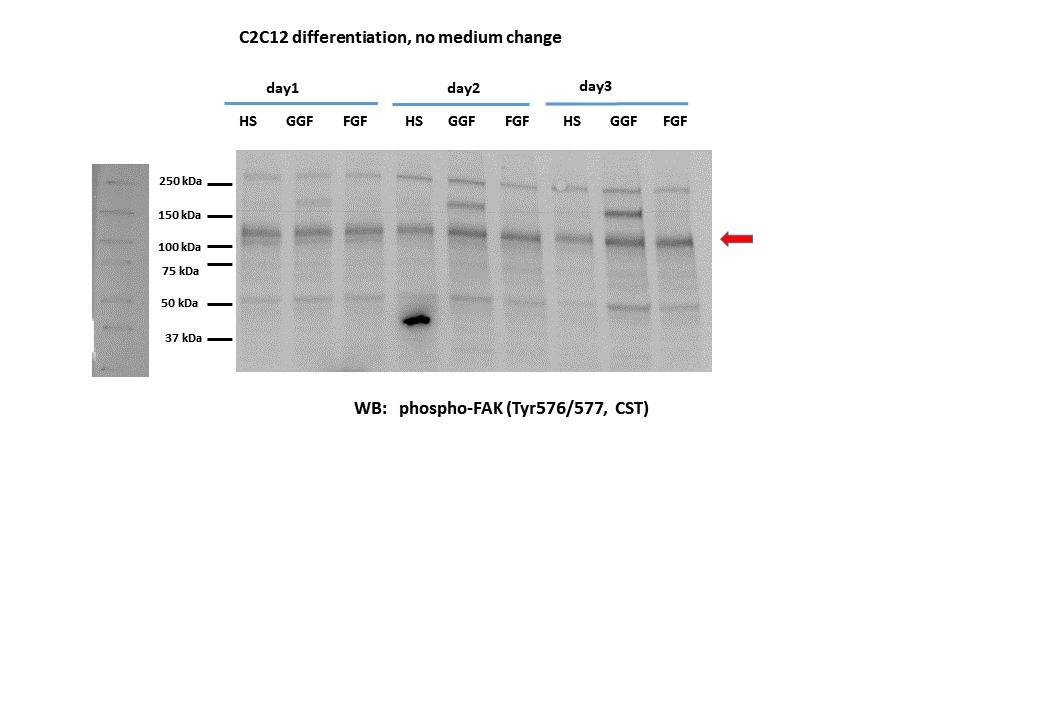

Supplement: Supplementary file 1 [file biology-11-00682-s001.zip › Figure S13.PNG]

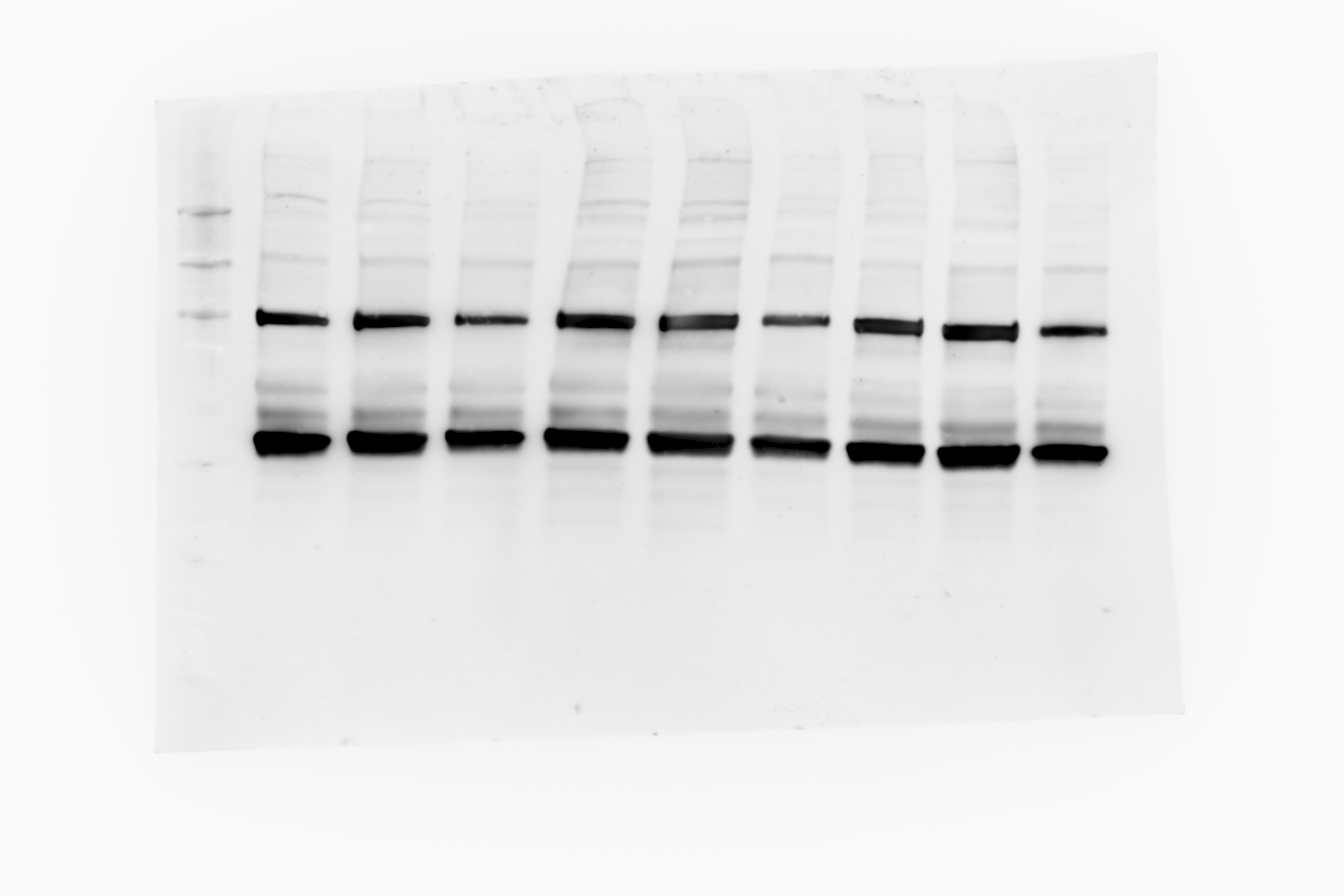

Supplement: Supplementary file 1 [file biology-11-00682-s001.zip › Figure S14.tiff]

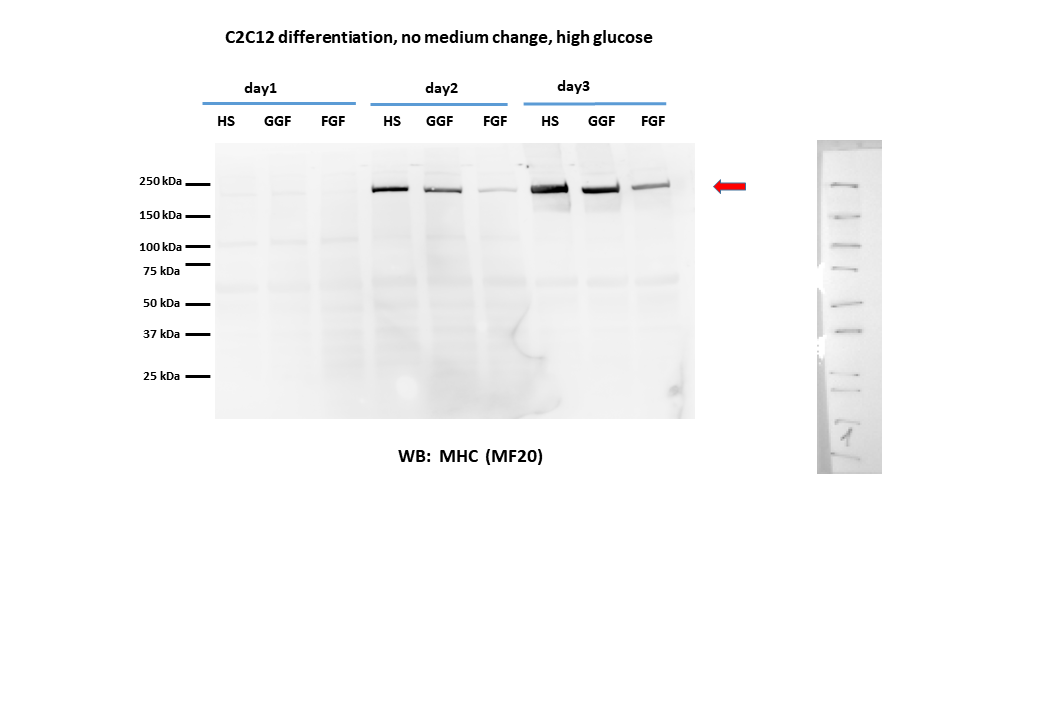

Supplement: Supplementary file 1 [file biology-11-00682-s001.zip › Figure S8.PNG]

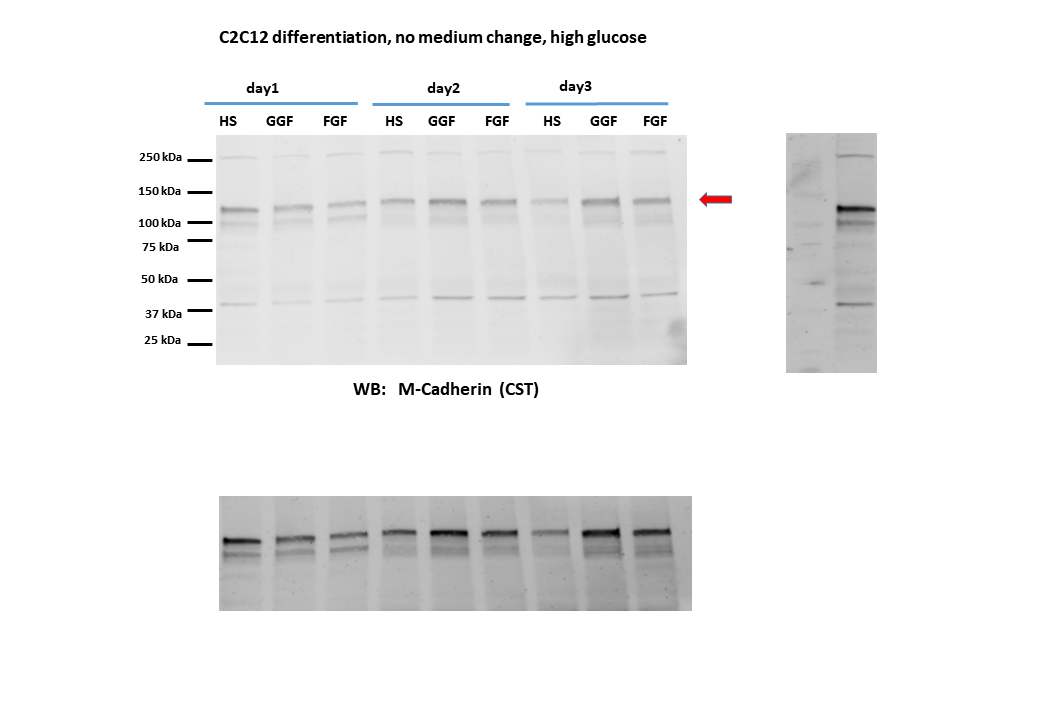

Supplement: Supplementary file 1 [file biology-11-00682-s001.zip › Figure S9.PNG]
